# Busting MisconSEXions: evaluation of a social media knowledge translation initiative addressing myths about sex

**DOI:** 10.3389/fpsyg.2024.1347493

**Published:** 2024-06-28

**Authors:** Kiarah M. K. O’Kane, Simone Y. Goldberg, Katrina N. Bouchard, Samantha J. Dawson

**Affiliations:** ^1^Department of Psychology, The University of British Columbia, Vancouver, BC, Canada; ^2^Department of Obstetrics and Gynaecology, The University of British Columbia, Vancouver, BC, Canada

**Keywords:** sexuality, sex education, sexual knowledge, knowledge translation, implementation science, social media, gender, sexual orientation

## Abstract

There is a critical gap in sex education such that many people lack access to evidence-based and accessible information about sexuality, putting them at risk for endorsing myths about sex and in turn having poorer sexual wellbeing. To address this gap, we developed a novel social media knowledge translation initiative—MisconSEXions—to debunk common myths about sexuality. The goal of this study was twofold. First, to examine whether exposure to MisconSEXions is effective for reducing sexuality myth endorsement. Second, to evaluate the acceptability (participants’ satisfaction with the content), appropriateness (the perceived fit of the content with participants), adoption (participants’ intention to engage with the initiative), and penetration (participants’ perception of the content’s impact on their lives) of MisconSEXions among study participants. We also examined possible group differences in our observed effects by assigned sex, gender modality, and sexual orientation. A large and diverse sample (*N* = 2,356) of adults completed an online survey and reported on their demographics, sexuality myth endorsement before and after exposure to MisconSEXions content, and the acceptability, appropriateness, adoption, and penetration of the MisconSEXions content. We found that participants’ sexuality myth endorsement was significantly lower following exposure to MisconSEXions content, and this effect held across assigned sex, gender modality, and sexual orientation groups. Regardless of participants’ assigned sex, gender modality, or sexual orientation, MisconSEXions content was reported to be both acceptable and appropriate to people’s lives. Participants reported relatively low levels of adoption, such that they reported reluctance to engage with the content on social media. Additionally, participants reported mixed feelings regarding the impact of the content on their lives (i.e., penetration). Overall, the findings have implications for how sexuality social media knowledge translation initiatives can fill important gaps in providing inclusive and accessible sex education.

## 1 Introduction

People often lack access to sex education or receive misinformation about sexuality. This is problematic because receiving limited or inaccurate information about sex is linked with endorsing myths about sexuality (Erbil, 2019) and in turn with poorer sexual outcomes (Baker and De Silva, 1988; Nobre and Pinto-Gouveia, 2006; Peixoto and Nobre, 2014; Erbil, 2019; Bakay et al., 2021). Thus, developing and testing novel methods to disseminate accurate information about sexuality is critical. Social media is a unique method of reaching wide and diverse audiences (Chan et al., 2020; Lu et al., 2021), and may have utility as a sex education tool. However, limited research has examined whether social media can be used to increase general sexual knowledge, rather than address specific sexual difficulties (Rosen and Brotto, 2021). Thus, the goal of the present study was to develop and test a sexuality social media knowledge translation initiative—MisconSEXions—that busts myths about sexuality using empirical evidence. Specifically, we examined whether exposure to MisconSEXions was associated with reductions in sexuality myth endorsement. We also explored whether MisconSEXions was acceptable and appropriate to participants, whether they reported that they would engage with the content on Instagram (adoption), and whether they perceived that this content impacted their lives (penetration).

### 1.1 Sex education and sexuality myth endorsement

People often receive limited or inaccurate education about sexuality, which in part stems from sex education curricula being non-comprehensive and unstandardized (Youth Co, 2018; SIECCAN, 2019; Action Canada, 2020; Narushima et al., 2020; Pampati et al., 2021; Wood et al., 2021). Approximately 50% of North American adolescents report receiving sex education that meets minimum international standards [i.e., only teaching about birth control methods and preventing sexually transmitted infections (STIs); Action Canada, 2020; Guttmacher Institute, 2022]. Outside of school settings, many people seek out information about sexuality from family, peers, or media (Evcili and Golbasi, 2017; Yeo and Chu, 2017), but these sources may be biased or provide inaccurate information (Ward, 2003; Coyne et al., 2019; Sartin-Tarm et al., 2021; Papp et al., 2022; Ward et al., 2022a,b). Receiving accurate information about sexuality in educational settings has a range of benefits for health (e.g., lower STI rates), social (e.g., greater empathy), and sexual (e.g., greater sexual communication) outcomes (Lo Presto et al., 1985; Constantine et al., 2015; Haberland and Rogow, 2015; Goldfarb and Lieberman, 2021; Ross et al., 2021; Ünal Toprak and Turan, 2021; Akalin, 2022; Ma et al., 2022; Hu et al., 2023). Moreover, the benefits of sex education on these outcomes persist over time (Morales et al., 2020), demonstrating the critical importance of addressing gaps in the accessibility of information about sexuality.

In addition to the general lack of access to comprehensive sex education, there are specific disparities in who receives *relevant* sex education based on their identities, including assigned sex, gender modality^1^ [i.e., whether individuals’ current gender identity aligns with the gender/sex they were assigned at birth (cisgender) or does not align (transgender and gender-diverse)], and sexual orientation. Sex education curricula are often gender/sex-segregated (Charmaraman et al., 2012; Hobaica and Kwon, 2017; Hobaica et al., 2019), or primarily focus on people with majoritized identities (i.e., those with greater structural/institutional power: assigned male at birth, cisgender, heterosexual; van Anders et al., 2022) versus minoritized identities (i.e., those with less structural/institutional power: assigned female at birth, transgender and gender-diverse, LGBQ+; Steinke et al., 2017; Rabbitte, 2020; Garg and Volerman, 2021; Pampati et al., 2021; Delmonaco and Haimson, 2022; Jia et al., 2022; Epps et al., 2023). For people with minoritized identities, failing to receive relevant sex education is associated with negative health (e.g., greater symptoms of depression), social (e.g., greater peer victimization), and sexual (e.g., greater sexual abuse victimization) outcomes (Gowen and Winges-Yanez, 2014; Bodnar and Tornello, 2019; Charley et al., 2023). Conversely, comprehensive sex education benefits LGBTQ+ youth’s mental health and is associated with less peer victimization (Blake et al., 2001; Greytak et al., 2013; Constantine et al., 2015; Proulx et al., 2019; Goldfarb and Lieberman, 2021). For people assigned female at birth, comprehensive sex education is linked with greater self-efficacy for experiencing sexual pleasure (Warshowsky et al., 2020). As such, receiving comprehensive sex education is beneficial for minoritized groups’ overall wellbeing. However, it is not yet well understood whether receiving comprehensive information about sexuality has differing degrees of benefit for minoritized and majoritized groups.

Having accurate sexual knowledge, which is facilitated through sex education, is important because greater endorsement of sexual myths is associated with poorer sexual wellbeing (e.g., higher sexual distress, lower sexual satisfaction, and lower sexual function; Baker and De Silva, 1988; Nobre and Pinto-Gouveia, 2006; Peixoto and Nobre, 2014; Erbil, 2019; Bakay et al., 2021). Despite being more likely to receive sex education relevant to their identities in school, majoritized groups (i.e., cisgender and heterosexual people, people assigned male at birth) are at greater risk of endorsing sexual myths than minoritized groups (Mosher, 1979; Eisenberg et al., 2004; Kukulu et al., 2009; Ziherl and Masten, 2010; Peixoto and Nobre, 2014; Evcili and Golbasi, 2017; Ekrem et al., 2022). This may be because majoritized groups are less likely than minoritized groups to seek out additional information about sexuality beyond what they learned in school to fill gaps in their sexual knowledge (Charest et al., 2016; Steinke et al., 2017).

The Information Motivation Behavioral Skills (IMB) Model provides theoretical context as to why exposure to accurate sexual information may contribute to changes in sexuality myth endorsement and in turn have downstream benefits for sexual wellbeing. The IMB Model posits that if people are exposed to accurate information and are motivated to utilize the information, they can enact the skills needed for behavioral change (Fisher and Fisher, 1992). Applied to sexuality, if people receive accurate comprehensive education about sexuality and are motivated to engage with the content, they may be better equipped to revise their inaccurate beliefs and change their approach to sex as well. For example, if people receive accurate education about masturbation, people who were reluctant to masturbate may use this new information to change their sexual behaviors to include masturbation. This addition to their sexual repertoire could have benefits for their sexual wellbeing such as greater sexual satisfaction or lower sexual distress (Carvalheira and Leal, 2013; Coleman and Bockting, 2013).

### 1.2 Social media for sexuality knowledge translation

There is a “valley of death” or profound gap in translating scientific findings into practice (Canadian Institutes of Health Research, 2011), such that new findings take approximately 17 years to be implemented into practice (Green, 2008), including sex education curricula. Knowledge translation is the practice of moving research findings into practice—whether through implementing scientific findings into policy, or individuals implementing findings into their lives (Jacobson et al., 2003; Estabrooks et al., 2006; Graham et al., 2006, 2007; Proctor et al., 2011; Straus et al., 2011; Grimshaw et al., 2012). One way to address the knowledge-to-practice gap in sex education is through developing innovative and accessible knowledge translation methods to disseminate sexuality science. Social media is one such avenue for knowledge translation of updated, inclusive, and evidence-based sex education to large and diverse audiences (Hamm et al., 2013; Perrin, 2015; Chan et al., 2020; Lu et al., 2021). Furthermore, given that misinformation often proliferates on social media, combatting inaccurate sexual information with evidence-based findings is important for promoting sexual knowledge.

Initial evidence suggests that sexuality knowledge translation initiatives can change individuals’ beliefs and attitudes about sexuality, and in turn, their sexual behaviors. Exposure to sexual health information via social media knowledge translation initiatives is associated with increases in knowledge about STIs and contraception (Guse et al., 2012; Wadham et al., 2019), and in turn with behavioral outcomes, like increased frequency of STI testing, condom use, and contraception use (Bull et al., 2012; Gabarron and Wynn, 2016; Stevens et al., 2017). Social media knowledge translation initiatives are also efficacious for improving knowledge about specific sexual problems or gender diversity, including bolstering new and expectant parents’ confidence in discussing sexual concerns (Rosen et al., 2021), improving knowledge about gender/sex (Beischel et al., 2021), and increasing knowledge about specific sexual difficulties (Jackowich et al., 2022). To date, knowledge translation research has primarily focused on sexual health or specific sexual difficulties, as opposed to general sexual knowledge. Thus, a key gap in the literature is evaluating whether sexuality social media knowledge translation initiatives improve knowledge about a broad range of sexuality domains.

In addition to evaluating impacts on sexual knowledge, evaluating how successfully sexuality social media knowledge translation initiatives are implemented sheds light on individuals’ experiences with the initiatives. In their taxonomy of implementation outcomes, Proctor et al. (2011) describe several implementation targets, including acceptability (i.e., how satisfactory the initiative is to individuals), appropriateness (i.e., the perceived fit of the initiative with individuals), adoption (i.e., individuals’ intention to engage with the initiative), and penetration (i.e., individuals’ perception of the initiative’s impact on them). Initial evidence suggests that sexuality social media knowledge translation initiatives garner positive impressions on implementation outcomes (Brotto et al., 2021; Rosen et al., 2021; Jackowich et al., 2022; Lavery et al., 2023). Importantly though, individuals express reluctance to adopt sexuality social media initiatives (e.g., publicly engage or be affiliated with them; Jackowich et al., 2022). Thus, while sexuality social media knowledge translation initiatives may be well received by users, the public nature of social media may result in less adoption.

There are group-level differences in social media usage for sex education and receptivity to social media platforms in general that may similarly impact receptivity to sexuality-related social media knowledge translation initiatives. LGBTQ+ people seek out information about sexuality on social media more frequently than cisgender and heterosexual individuals do (Ceglarek and Ward, 2016; Charest et al., 2016; Herrmann et al., 2023). Additionally, with respect to general social media use, people assigned female at birth use Instagram more frequently and with more active engagement (e.g., “liking,” commenting on, and sharing posts) than people assigned male at birth (Gil de Zúñiga et al., 2017; Vaid and Harari, 2021; Laor, 2022; Legkauskas and Kudlaitė, 2022). Therefore, minoritized groups may respond more positively to sexuality knowledge translation initiatives delivered via Instagram than majoritized groups given their use of social media for sex education or greater overall receptivity to Instagram.

### 1.3 Present study

The present study aimed to evaluate whether exposure to MisconSEXions content would reduce individuals’ sexuality myth endorsement. We hypothesized that majoritized groups (by assigned sex, gender modality, and sexual orientation) would have greater reductions in their sexuality myth endorsement after exposure to MisconSEXions content than minoritized groups. We also aimed to evaluate the implementation of MisconSEXions content, including potential group differences in these outcomes. In general, we hypothesized that MisconSEXions content would have relatively high scores for acceptability and appropriateness, with lower scores for adoption and penetration. Based on the findings that people assigned male at birth, cisgender people, and heterosexual people are less likely to use Instagram or access education about sexuality on social media (Ceglarek and Ward, 2016; Charest et al., 2016; Gil de Zúñiga et al., 2017; Vaid and Harari, 2021; Laor, 2022; Legkauskas and Kudlaitė, 2022; Herrmann et al., 2023), we hypothesized that cisgender heterosexual men (i.e., majoritized groups) relative to other groups would report lower scores for implementation outcomes.

## 2 Materials and methods

### 2.1 Participants

Participants included undergraduate students at the University of British Columbia recruited through the undergraduate psychology subject pool and community members recruited through social media advertisements (e.g., Reddit). Recruitment through social media involved posting advertisements in groups or subreddits after contacting moderators of groups relevant to the survey topic (i.e., r/sex) and obtaining permission to post. Undergraduate students were compensated with 0.5 course credits per each half hour of participation in the study, while community participants were not compensated for their participation. All procedures were approved by the Behavioural Research Ethics Board at the University of British Columbia.

To be eligible for the study, participants had to be over 18 years of age, fluent in English, and have access to a device that could connect to the Internet. Recruitment of community members was open to individuals living in any country. A total of 1,334 undergraduate participants and 2,585 community members signed up for and consented to participate in the study. Of those who consented, participants were removed prior to analysis for the following reasons: did not start the survey (*n* = 54 and *n* = 368, undergraduate and community members respectively), did not view any MisconSEXions content (*n* = 15 and *n* = 37), did not complete any of the core measures (e.g., sexuality myth endorsement pre- and post-MisconSEXions or implementation outcomes; *n* = 15 and *n* = 724), failed two or more of three attention checks (*n* = 13 and *n* = 265), or indicated that we should not use their data in analyses based on a question at the end of the survey (*n* = 60 and *n* = 12). Removal of these participants left a sample of 1,177 undergraduate students and 1,179 community members for a total sample of 2,356 participants.

### 2.2 Materials

#### 2.2.1 MisconSEXions

The research team developed an Instagram-based knowledge translation initiative in 2021 designed to bust myths about sexuality using empirical information. Each post on MisconSEXions was written by the research team and reviewed by the study’s Principal Investigator. The posts were written in lay language and aimed to be inclusive with respect to gender/sex and sexual orientation. Each post addressed a specific myth from a broad range of topics about sexuality. Within each post, the first slide was titled “Myth,” and described the myth being debunked within the post. Subsequent slides were titled “Fact,” and included empirical evidence to debunk the myth. Information for the “Fact” slides relied on findings from recent (2016-present), well-powered studies (e.g., meta-analyses and population studies), and studies sampling people with diverse gender identities and sexual orientations. Citations to studies supporting the information on “Fact” slides were included in post captions. The posts included colorful and inclusive visual graphics corresponding to the topic of the myth (e.g., an illustration of a banana for a post about erections). Posts were posted on the MisconSEXions Instagram account (@misconsexions), and relevant hashtags were used to help individuals looking for sex education content find the posts. Posts were made at a rate of approximately five posts per month. By November 2023, the MisconSEXions Instagram account had amassed over 1,000 followers. An example of a MisconSEXions post is accessible on the Open Science Framework (OSF), along with all other measures, materials, and data.^2^

### 2.3 Measures

#### 2.3.1 Demographics

Participants reported on their demographic characteristics, including age, assigned sex, gender modality (i.e., whether participants were cisgender or transgender/gender-diverse), sexual orientation, race/ethnicity/nationality, and whether they received sex education in school.

##### 2.3.1.1 Gender modality

Participants were grouped into either a majoritized, cisgender group or a minoritized, transgender and gender-diverse group. The transgender and gender-diverse group included participants who identified as transgender and those who reported gender-diverse identities (e.g., non-binary).

##### 2.3.1.2 Sexual orientation

Participants were grouped into either a majoritized sexual orientation group or a minoritized sexual orientation group. Participants in the majoritized group were those who selected the heterosexual response option, and participants in the minoritized group were those who selected any other sexual orientation (e.g., lesbian, gay, bisexual, queer; LGBQ+).

#### 2.3.2 Sexual myths

Participants were presented with 10 statements that were myths about sexuality (e.g., “people can masturbate too much”). Myths were introduced to participants as statements about sexuality commonly endorsed by people in society. The 10 sexual myths were selected because they were the most highly endorsed in a previous study. Participants were asked to rate their degree of agreement with the 10 statements on a 5-point Likert scale from 1 (*strongly disagree*) to 5 (*strongly agree*), or 0 (*I don’t know*). Two average myth endorsement scores were calculated for each participant by averaging endorsement ratings for each of the 10 myths before (pre) and after (post) exposure to MisconSEXions. Higher scores represent greater average myth endorsement (possible range = 1.00–5.00). Responses of 0 (*I don’t know*) to individual myths were excluded from the calculation of pre-MisconSEXions and post-MisconSEXions myth endorsement scores as they represent a lack of knowledge rather than a degree of myth endorsement. Average pre- or post-MisconSEXions myth endorsement scores were not created for participants missing over 20% of data for individual myths (*n* = 11).

#### 2.3.3 Implementation outcomes

Participants completed a researcher-derived measure of implementation outcomes based on the Proctor et al. (2011) taxonomy and measures of implementation outcomes used in studies evaluating sexuality knowledge translation initiatives (Rosen et al., 2021; Jackowich et al., 2022). Participants rated the acceptability, appropriateness, adoption, and penetration of MisconSEXions content. Higher scores represent more positive impressions of MisconSEXions content (possible range = 1.00–5.00).

##### 2.3.3.1 Acceptability

Participants reported on how acceptable they found MisconSEXions content to be using five face-valid items (e.g., “How understandable was the content?”). Participants responded to items on a 5-point Likert scale with scale anchors of 1 (*not at all/not at all good*), 3 (*somewhat/somewhat good*), and 5 (*very much/very good*), depending on the question.

##### 2.3.3.2 Appropriateness

The perceived appropriateness of MisconSEXions content was measured using three face-valid items. Two items asked how appropriate the content was for participants’ own lives (e.g., “How relevant was the content to your life?”), and one item asked participants their perspective on the appropriateness of the mode of delivery used for sharing MisconSEXions content (i.e., “How suitable do you think Instagram is for sharing this content about sex and sexuality?”). Participants responded to items using a 5-point Likert scale with scale anchors of 1 (*not at all/not at all suitable*), 3 (*somewhat/somewhat suitable*), and 5 (*very much/very suitable*), depending on the question.

##### 2.3.3.3 Adoption

Participants reported on the extent to which they would engage with MisconSEXions on Instagram using four face-valid items (e.g., “How likely would you be to follow MisconSEXions on Instagram?”). Participants responded to items using a 5-point Likert scale with scale anchors of 1 (*not at all likely*), 3 (*somewhat likely*), and 5 (*very likely*).

##### 2.3.3.4 Penetration

Participants’ perceptions of how much MisconSEXions content impacted them were measured using five face-valid items (e.g., “How much did the content change your attitudes about the topics that were discussed?”). Participants responded to items on a 5-point Likert scale with scale anchors of 1 (*not at all/not at all interested*), 3 (*somewhat/somewhat interested*), and 5 (*very much/yes, a lot/very interested*), depending on the question.

### 2.4 Procedure

Undergraduate participants received a secure link to an online consent form after signing up to participate in the study through the undergraduate psychology subject pool. Participants in the community sample accessed the online consent form via a secure link in the advertisement posted on social media (e.g., on Reddit). After consenting via an online consent form, participants were directed to complete an online survey hosted on the Qualtrics platform. Participants then reported on their demographic information, and their agreement with the 10 statements (myths) about sexuality (pre-MisconSEXions exposure). Next, participants were exposed to a list of 40 MisconSEXions posts organized by topic (i.e., desire, arousal, masturbation, sexual activity, orgasm, genitals, and sexual response cycles) and were instructed to view the posts. Participants were able to click on as few or as many posts as they wished to and read the post content. When participants clicked on a post, a hyperlink opened a new tab in their browser with a PDF file containing the post for participants to view. The survey page with the 40 posts remained locked for 3 min to prevent participants from immediately skipping through the MisconSEXions content, and participants could only proceed in the survey after the 3 min passed. Following exposure to MisconSEXions content, participants completed measures reporting on implementation outcomes. Lastly, participants reported on their agreement with the *same* 10 myths about sexuality that they reported on prior to viewing MisconSEXions content (post-MisconSEXions exposure). Following their completion of the survey, participants were directed to an online debriefing form, and those in the undergraduate sample received course credit as compensation.

### 2.5 Data analysis

We used SPSS version 28.0 to conduct all analyses. Participants who did not have both a pre- and post-MisconSEXions average myth endorsement score (*n* = 247; including the 11 who were missing over 20% of individual myth data and thus did not have average myth endorsement scores and 236 who skipped the sexual myths measure entirely) were excluded from the analysis testing whether exposure to MisconSEXions content reduced myth endorsement, but were included in the implementation outcomes analyses because they did report viewing the MisconSEXions content. A 2 (*Time*: pre-MisconSEXions, post-MisconSEXions) × 2 (*Assigned sex*: male, female) × 2 (*Gender modality*: cisgender, transgender and gender-diverse) × 2 (*Sexual orientation*: heterosexual, LGBQ+) mixed-model Analysis of Variance (ANOVA) was used to test the hypothesis that there would be a change in average myth endorsement after exposure to MisconSEXions content and that the degree of change would differ as a function of group (assigned sex, gender modality, and sexual orientation).

For the MisconSEXions implementation outcomes, a 2 (*Assigned sex*: male, female) × 2 (*Gender modality*: cisgender, transgender and gender-diverse) × 2 (*Sexual orientation*: heterosexual, LGBQ+) factorial ANOVA was used for each implementation outcome item to test the hypothesis that majoritized vs. minoritized groups would differ in their scores on implementation outcomes. Separate ANOVAs were conducted for individual implementation outcome items because items were moderately positively correlated with one another, suggesting that they captured related but distinct experiences of MisconSEXions content.

## 3 Results

### 3.1 Participant characteristics

Our sample was diverse with respect to gender modality, sexual orientation, and race/ethnicity/nationality. See Table 1 for information about participant demographics. In the sample, 76.4% of participants reported being Instagram users (i.e., downloaded the Instagram app). Of the participants who reported being Instagram users, in a typical week, 21.6% reported using Instagram between one to five times per week, 12.9% reported using it once per day, and 41.5% reported using it more than once per day. The remaining 24.0% reported no Instagram use during a typical week despite having downloaded the app.

**TABLE 1:** Participant demographics.

Participant characteristics	Undergraduate sample	Community sample
	*n* (%) or *M* (SD)	*n* (%) or *M* (SD)
Age (years)	20.80 (3.97)	29.29 (11.10)
**Assigned sex**		
Female	961 (81.6%)	656 (55.6%)
Male	216 (18.4%)	523 (44.4%)
**Gender modality**		
Cisgender	1,132 (96.2%)	968 (82.1%)
Transgender and gender-diverse	45 (3.8%)	211 (17.9%)
**Sexual orientation**		
Heterosexual	823 (69.9%)	523 (44.4%)
LGBQ+, including^a^	354 (30.1%)	656 (55.6%)
Asexual	49 (4.2%)	115 (9.8%)
Bisexual	177 (15.0%)	275 (23.3%)
Gay	17 (1.4%)	36 (3.1%)
Lesbian	19 (1.6%)	40 (3.4%)
Pansexual	36 (3.1%)	68 (5.8%)
Queer	38 (3.2%)	63 (5.3%)
Not listed/unspecified^b^	18 (1.5%)	59 (5.0%)
**Race/ethnicity/nationality**		
African	7 (0.6%)	5 (0.4%)
Black	1 (0.1%)	9 (0.8%)
Biracial/multiracial	48 (4.1%)	49 (4.2%)
Caribbean	1 (0.1%)	2 (0.2%)
East Asian	428 (36.4%)	37 (3.1%)
European	85 (7.2%)	335 (28.4%)
Indigenous (First Nations, Métis, Inuit, Native American, Aboriginal, Alaska Native)	8 (0.7%)	11 (0.9%)
Latino/Latina	27 (2.3%)	37 (3.1%)
Middle Eastern	35 (3.0%)	16 (1.4%)
North American	268 (22.7%)	526 (44.8%)
South Asian	134 (11.4%)	49 (4.2%)
Southeast Asian	93 (7.9%)	32 (2.7%)
Not listed/unspecified^c^	42 (3.5%)	68 (5.8%)
**Received sex education in school**		
Yes	883 (75.0%)	852 (72.2%)
No	209 (17.8%)	246 (20.9%)
Unsure	85 (7.2%)	81 (6.9%)

^a^The percentages shown for specific sexual orientation identities (e.g., asexual) were calculated based on the total number of participants in each sample (*N* = 1,177 and 1,179, respectively).

^b^Additional LGBQ+ identities reported were bicurious, demisexual, graysexual, polysexual, and questioning.

^c^Additional race/ethnicity/nationality identities reported were Australian, British, Caucasian, Jewish, and White.

### 3.2 Myth endorsement

The four-way interaction between time, assigned sex, gender modality, and sexual orientation was not significant, *F*(1, 2,101) = 0.86, *p* = 0.35, partial η^2^ = 0.000. The three-way interactions between time, assigned sex, and gender modality, *F*(1, 2,101) = 0.51, *p* = 0.47, partial η^2^ = 0.000, time, assigned sex, and sexual orientation, *F*(1, 2,101) = 1.46, *p* = 0.23, partial η^2^ = 0.001, and time, gender modality, and sexual orientation, *F*(1, 2,101) = 0.23, *p* = 0.63, partial η^2^ = 0.000 were not significant. The two-way interactions between time and assigned sex, *F*(1, 2,101) = 0.46, *p* = 0.50, partial η^2^ = 0.000, time and gender modality, *F*(1, 2,101) = 0.58, *p* = 0.44, partial η^2^ = 0.000, and time and sexual orientation, *F*(1, 2,101) = 0.21, *p* = 0.65, partial η^2^ = 0.000 were not significant.

There was a significant main effect of time, *F*(1, 2,101) = 107.59, *p* < 0.001, partial η^2^ = 0.049, such that participants’ average myth endorsement scores were lower post-MisconSEXions exposure (*M* = 2.62; SD = 0.58), compared to their average myth endorsement scores pre-MisconSEXions exposure (*M* = 3.26; SD = 0.48). This suggests that, on average, participants had significantly lower sexuality myth endorsement following exposure to MisconSEXions content.

### 3.3 Implementation outcomes

There were no significant three-way interactions (all *F*s ≤ 2.04, *p*s ≥ 0.15) or two-way interactions (all *F*s ≤ 2.46, *p*s ≥ 0.12) for any of the factorial ANOVAs for individual implementation outcome items. Of note, degrees of freedom vary throughout the implementation outcome results due to participant attrition through the implementation outcome section of the survey or participants skipping certain implementation outcome items.

#### 3.3.1 Acceptability

Five items were used to assess overall acceptability of MisconSEXions content. With regard to how much participants liked MisconSEXions posts, there were no significant main effects of assigned sex, *F*(1, 2,348) = 0.23, *p* = 0.63, partial η^2^ = 0.000, gender modality, *F*(1, 2,348) = 0.26, *p* = 0.61, partial η^2^ = 0.000, or sexual orientation, *F*(1, 2,348) = 0.06, *p* = 0.80, partial η^2^ = 0.000. Overall, participants reported liking MisconSEXions posts quite a bit (*M* = 3.93; SD = 0.91).

In terms of how participants rated the quality of information presented on MisconSEXions, there were no significant main effects of assigned sex, *F*(1, 2,347) = 0.03, *p* = 0.86, partial η^2^ = 0.000, gender modality, *F*(1, 2,347) = 0.95, *p* = 0.33, partial η^2^ = 0.000, or sexual orientation, *F*(1, 2,347) = 3.33, *p* = 0.07, partial η^2^ = 0.001. On average, participants rated the quality of information on MisconSEXions as quite good (*M* = 4.17; SD = 0.87).

With respect to how understandable MisconSEXions content was, there were no significant main effects of assigned sex, *F*(1, 2,346) = 0.02, *p* = 0.89, partial η^2^ = 0.000, gender modality, *F*(1, 2,346) = 0.08, *p* = 0.78, partial η^2^ = 0.000, or sexual orientation, *F*(1, 2,346) = 0.001, *p* = 0.97, partial η^2^ = 0.000. On average, participants reported that they understood MisconSEXions content very much (*M* = 4.66; SD = 0.61).

In terms of how visually appealing MisconSEXions content was, there were no significant main effects of assigned sex, *F*(1, 2,347) = 3.56, *p* = 0.06, partial η^2^ = 0.002, gender modality, *F*(1, 2,347) = 0.12, *p* = 0.73, partial η^2^ = 0.000, or sexual orientation, *F*(1, 2,347) = 0.40, *p* = 0.52, partial η^2^ = 0.000. Participants thought MisconSEXions content was quite visually appealing (*M* = 3.96; SD = 0.99).

Lastly, with regard to how inclusive participants found MisconSEXions content to be (i.e., with respect to gender/sex, age, bodies, sexual orientation, etc.), there were no significant main effects of assigned sex, *F*(1, 2,344) = 1.97, *p* = 0.16, partial η^2^ = 0.001, or sexual orientation, *F*(1, 2,344) = 1.26, *p* = 0.26, partial η^2^ = 0.001. There was a significant main effect of gender modality, *F*(1, 2,344) = 6.21, *p* = 0.01, partial η^2^ = 0.003. Both gender modality groups perceived that MisconSEXions content was quite inclusive; however, cisgender participants perceived that MisconSEXions content was slightly more inclusive (*M* = 4.36; SD = 0.83) than transgender and gender-diverse participants did (*M* = 4.18; SD = 0.95).

#### 3.3.2 Appropriateness

Appropriateness of MisconSEXions content was assessed with three items. In terms of how relevant participants found MisconSEXions content to be to their lives, there were no significant main effects of assigned sex, *F*(1, 2,344) = 2.38, *p* = 0.12, partial η^2^ = 0.001, or sexual orientation, *F*(1, 2,344) = 0.006, *p* = 0.94, partial η^2^ = 0.000. There was a significant main effect of gender modality, *F*(1, 2,344) = 4.43, *p* = 0.04, partial η^2^ = 0.002. Both gender modality groups found MisconSEXions content to be somewhat relevant to their lives; however, cisgender participants perceived that MisconSEXions content was slightly more relevant (*M* = 3.49; SD = 1.09) than transgender and gender-diverse participants did (*M* = 3.13; SD = 1.09).

With respect to how helpful participants found MisconSEXions content to be for them personally, there were no significant main effects of assigned sex, *F*(1, 2,346) = 0.36, *p* = 0.55, partial η^2^ = 0.000, or sexual orientation, *F*(1, 2,346) = 0.24, *p* = 0.63, partial η^2^ = 0.000. There was a significant main effect of gender modality, *F*(1, 2,346) = 9.23, *p* = 0.002, partial η^2^ = 0.004. While both gender modality groups found MisconSEXions content to be somewhat helpful, cisgender participants found MisconSEXions content to be slightly more helpful (*M* = 3.40; SD = 1.13) than transgender and gender-diverse participants did (*M* = 2.95; SD = 1.14).

Lastly, for how suitable participants thought Instagram was for sharing content about sex and sexuality, there were no significant main effects of gender modality, *F*(1, 2,330) = 0.02, *p* = 0.90, partial η^2^ = 0.000, or sexual orientation, *F*(1, 2,330) = 0.86, *p* = 0.35, partial η^2^ = 0.000. There was a significant main effect of assigned sex, *F*(1, 2,330) = 4.02, *p* = 0.04, partial η^2^ = 0.002. While both assigned sex groups thought that Instagram was quite suitable for sharing content about sex and sexuality, people assigned female at birth perceived Instagram to be slightly more suitable (*M* = 3.93; SD = 1.09) than people assigned male at birth did (*M* = 3.57; SD = 1.17).

#### 3.3.3 Adoption

Four items were used to assess participants’ overall willingness to adopt MisconSEXions. In terms of how likely participants reported they would be to “like” a MisconSEXions post on Instagram, there were no significant main effects of gender modality, *F*(1, 2,274) = 0.65, *p* = 0.42, partial η^2^ = 0.000, or sexual orientation, *F*(1, 2,274) = 2.06, *p* = 0.15, partial η^2^ = 0.001. There was a significant main effect of assigned sex, *F*(1, 2,274) = 7.25, *p* = 0.007, partial η^2^ = 0.003, such that people assigned female at birth reported being somewhat likely to “like” a MisconSEXions post on Instagram (*M* = 2.99; SD = 1.40) compared to people assigned male at birth who reported being a little bit likely to “like” a post (*M* = 2.44; SD = 1.43).

With regard to participants’ reported likelihood of following MisconSEXions on Instagram, there were no significant main effects of gender modality, *F*(1, 2,274) = 1.03, *p* = 0.31, partial η^2^ = 0.000, or sexual orientation, *F*(1, 2,274) = 1.07, *p* = 0.30, partial η^2^ = 0.000. There was a significant main effect of assigned sex, *F*(1, 2,274) = 3.90, *p* = 0.048, partial η^2^ = 0.002, such that people assigned female at birth reported being somewhat likely to follow MisconSEXions on Instagram (*M* = 2.75; SD = 1.40) compared to people assigned male at birth who reported being a little bit likely to follow MisconSEXions (*M* = 2.27; SD = 1.40).

In terms of participants’ reported willingness to share a MisconSEXions post with other people, there were no significant main effects of assigned sex, *F*(1, 2,277) = 0.21, *p* = 0.64, partial η^2^ = 0.000, gender modality, *F*(1, 2,277) = 0.75, *p* = 0.39, partial η^2^ = 0.000, or sexual orientation, *F*(1, 2,277) = 1.05, *p* = 0.30, partial η^2^ = 0.000. On average, the sample reported being a little bit likely to share a MisconSEXions post with other people (*M* = 2.49; SD = 1.30).

Regarding participants’ reported likelihood of sharing information that they learned from MisconSEXions posts with other people, there were no significant main effects of assigned sex, *F*(1, 2,278) = 0.13, *p* = 0.72, partial η^2^ = 0.000, gender modality, *F*(1, 2,278) = 0.02, *p* = 0.90, partial η^2^ = 0.000, or sexual orientation, *F*(1, 2,278) = 0.60, *p* = 0.44, partial η^2^ = 0.000. On average, participants reported that they were somewhat likely to share the information they learned from MisconSEXions posts with other people (*M* = 3.38; SD = 1.25).

#### 3.3.4 Penetration

To assess the overall penetration of MisconSEXions content, five items were used. With respect to whether participants perceived that they learned new information from the MisconSEXions content, there were no significant main effects of assigned sex, *F*(1, 2,204) = 0.42, *p* = 0.52, partial η^2^ = 0.000, gender modality, *F*(1, 2,204) = 0.002, *p* = 0.96, partial η^2^ = 0.000, or sexual orientation, *F*(1, 2,204) = 1.75, *p* = 0.19, partial η^2^ = 0.001. On average, participants perceived that they learned quite a bit of new information from the content (*M* = 3.71; SD = 1.04).

In terms of whether participants were surprised by any of the information in MisconSEXions content, there were no significant main effects of assigned sex, *F*(1, 2,203) = 0.007, *p* = 0.94, partial η^2^ = 0.000, gender modality, *F*(1, 2,203) = 0.16, *p* = 0.68, partial η^2^ = 0.000, or sexual orientation, *F*(1, 2,203) = 2.22, *p* = 0.14, partial η^2^ = 0.001. On average, participants reported that they were somewhat surprised by information in MisconSEXions content (*M* = 3.03; SD = 1.19).

Regarding how much participants felt exposure to MisconSEXions content changed their attitudes about the topics that were discussed in posts, there were no significant main effects of assigned sex, *F*(1, 2,203) = 0.15, *p* = 0.70, partial η^2^ = 0.000, gender modality, *F*(1, 2,203) = 1.44, *p* = 0.23, partial η^2^ = 0.001, or sexual orientation, *F*(1, 2,203) = 0.83, *p* = 0.36, partial η^2^ = 0.000. On average, participants reported that MisconSEXions content somewhat changed their attitudes about topics discussed in the posts (*M* = 2.67; SD = 1.15).

With respect to how much participants felt MisconSEXions content changed their attitudes about sex and sexuality, there were no significant main effects of assigned sex, *F*(1, 2,203) = 0.04, *p* = 0.84, partial η^2^ = 0.000, gender modality, *F*(1, 2,203) = 1.08, *p* = 0.30, partial η^2^ = 0.000, or sexual orientation, *F*(1, 2,203) = 3.20, *p* = 0.07, partial η^2^ = 0.001. On average, participants reported that MisconSEXions content changed their attitudes about sex and sexuality a little bit (*M* = 2.39; SD = 1.14).

Lastly, with regard to whether participants reported being interested in learning more about sex and sexuality after viewing MisconSEXions content, there were no significant main effects of assigned sex, *F*(1, 2,203) = 0.40, *p* = 0.52, partial η^2^ = 0.000, or sexual orientation, *F*(1, 2,203) = 2.69, *p* = 0.10, partial η^2^ = 0.001. There was a significant main effect of gender modality, *F*(1, 2,203) = 5.51, *p* = 0.02, partial η^2^ = 0.002, such that cisgender people reported being quite interested in learning more about sex and sexuality after viewing MisconSEXions content (*M* = 3.60; SD = 1.11) compared to transgender and gender-diverse people who reported being somewhat interested (*M* = 3.36; SD = 1.18).

### 3.4 Participant engagement with MisconSEXions content

Most participants (74.2%) reported viewing between 1 and 10 MisconSEXions posts, with the remaining minority of participants viewing between 11 and 40 posts (23.6%) or no posts (2.2%; these participants were excluded from the MisconSEXions evaluation analyses). Of participants who viewed at least one MisconSEXions post, on average, participants spent 5.47 min (SD = 7.42) looking at MisconSEXions content. With respect to the characteristics of participants who viewed no MisconSEXions posts (*n* = 52), 59.6% reported being assigned female at birth and 40.4% reported being assigned male, 84.6% reported being cisgender and 15.4% reported being transgender and gender-diverse, and 59.6% reported a heterosexual sexual orientation and 40.4% reported a LGBQ+ sexual orientation. Of these participants who viewed no posts, 48.1% reported doing so because they did not have time, 26.9% because they reported already having a solid knowledge of sexuality, 23.1% because the content did not look relevant to them, and 19.2% because the content did not look interesting to them (percentages add up to over 100% because participants could select more than one response option). Participants also reported on how suitable they thought platforms other than Instagram would be for sharing MisconSEXions content (possible range = 1.00–5.00). On average, participants reported that Facebook was somewhat suitable (*M* = 3.10, SD = 1.23), whereas YouTube (*M* = 3.61, SD = 1.24), TikTok (*M* = 3.70, *SD* = 1.28), Twitter (*M* = 3.53, SD = 1.23), and a dedicated website (*M* = 4.40, SD = 0.96) were all quite suitable.

## 4 Discussion

In the present study, we evaluated a social media knowledge translation initiative—MisconSEXions. We found that brief exposure to MisconSEXions content resulted in a small but significant reduction in sexuality myth endorsement in a diverse sample of adults. Contrary to our hypotheses, there were no group differences in this effect by assigned sex, gender modality, or sexual orientation. With respect to implementation outcomes, participants reported that MisconSEXions content was acceptable and generally appropriate to their lives. They did, however, report some reluctance to adopt MisconSEXions (i.e., engage with it on Instagram). Additionally, average scores for penetration (i.e., the perceived impact of MisconSEXions content on participants) ranged from “a little bit” to “quite a bit,” suggesting participants perceived that MisconSEXions content had a variable influence on different aspects of their sexual knowledge and attitudes. Contrary to our hypotheses, for most implementation outcomes the groups did not differ significantly, and when they did the effect sizes were negligible to very small.

### 4.1 Impact of MisconSEXions content on reducing sexuality myth endorsement

Brief exposure to MisconSEXions content during our study led to a small reduction in participants’ sexuality myth endorsement. Consistent with predictions from the IMB Model (Fisher and Fisher, 1992), when participants engaged with accurate sexual education through MisconSEXions content, we saw shifts in their knowledge and beliefs about sexuality. Indeed, participants on average spent over 5 min looking at content and most participants viewed between 1 and 10 posts, suggesting they were motivated to engage with the content. Additionally, in the present study, participants could view any MisconSEXions posts that they wanted to—meaning participants did not necessarily view posts corresponding to the myths that they endorsed pre-MisconSEXions exposure. Our findings highlight that participants did not need to view content busting the specific myths they reported their endorsement of to experience reductions in their overall sexuality myth endorsement. As such, receiving *any* accurate sexual information appears to have benefits for sexuality myth endorsement that generalize across topics, regardless of the *specific* topics discussed in content that individuals view.

Contrary to our hypotheses, we did not find group differences in the degree of change in participants’ myth endorsement after exposure to MisconSEXions content, suggesting that brief exposure to it was similarly effective across groups. Despite some groups—people assigned male at birth, heterosexual people, cisgender people—being more likely to endorse sexual myths (Mosher, 1979; Eisenberg et al., 2004; Kukulu et al., 2009; Ziherl and Masten, 2010; Peixoto and Nobre, 2014; Evcili and Golbasi, 2017; Ekrem et al., 2022), our findings suggest that MisconSEXions content was similarly effective at reducing myth endorsement, regardless of initial levels of myth endorsement.

### 4.2 Implementation of MisconSEXions content

In general, participants found MisconSEXions content to be acceptable. Indeed, one of the major strengths of MisconSEXions content was that participants found it to be “quite” likeable, visually-appealing, of good quality, and “very much” understandable. While the effect of gender modality on inclusivity of the content was significant, the effect size was very small, and the means revealed that both gender modality groups reported that MisconSEXions content was “quite” inclusive. The high acceptability scores in our sample highlight the success of intentionally designing inclusive sexuality social media knowledge translation initiatives to be satisfying regardless of individuals’ identities with respect to the graphics and language. Given that many individuals report dissatisfaction with the non-comprehensive sex education they received in school (Allen, 2005; Rutledge et al., 2011; Cense et al., 2020), our findings suggest that social media knowledge translation initiatives can be an appealing alternative avenue for comprehensive sex education that is inclusive and satisfactory to diverse groups of people.

While participants generally found Instagram to be an appropriate platform for hosting MisconSEXions content, our findings suggest that content could potentially be made more appropriate. Despite finding that the content was “quite” inclusive, participants reported that the content was only “somewhat” personally relevant and helpful. This suggests that MisconSEXions content could be improved with regard to appropriateness by tailoring the content even more. In our efforts to be broadly inclusive, MisconSEXions posts generally focused on busting myths applicable to most people, rather than myths endorsed by specific groups. However, people perceive sex education to be more relevant when it discusses topics pertaining to their identities (Gowen and Winges-Yanez, 2014; Hobaica et al., 2019; Tordoff et al., 2021; Epps et al., 2023). Indeed, sexuality social media knowledge translation initiatives focused on specific problems and populations have produced higher ratings of appropriateness (Rosen et al., 2021; Jackowich et al., 2022). As such, a downside of our goal to make MisconSEXions content broadly inclusive is that not all content will feel personally relevant to everyone; at the same time, no one group will be excluded from content. Indeed, one of the strengths of MisconSEXions content was that it appealed to a diverse audience and thus filled critical gaps in the accessibility of sex education for all groups.

Similar to other studies evaluating sexuality social media knowledge translation initiatives (Jackowich et al., 2022), our sample reported relatively low levels of adoption. Despite finding Instagram to be “quite” suitable for hosting MisconSEXions content, participants assigned male at birth and female at birth reported being only “a little bit” or “somewhat” likely respectively to follow the MisconSEXions account or “like” its posts on Instagram. Individuals report being wary of publicly affiliating themselves with sex education accounts that contain explicit sexual language or imagery (e.g., drawings of sex toys and genitals) due to social taboo (Ramallo et al., 2015; Jackowich et al., 2022). While participants of all groups reported being only “a little bit” likely to share a MisconSEXions *post* with other people, they did report being “somewhat” likely to share *information* they learned from MisconSEXions with others. This could suggest that participants’ reluctance to engage with MisconSEXions on Instagram centers around how explicitly and publicly the sexual information is presented, as opposed to the sexual information itself. Indeed, individuals report preferring sex education that can be accessed anonymously and privately (Selkie et al., 2011). Thus, the public nature of social media could be one potential barrier to the adoption of sexuality social media knowledge translation initiatives. However, it is possible that participants need not publicly engage with content (e.g., “liking” and sharing posts) to benefit from the knowledge. Indeed, in the present study, simply viewing posts for approximately 5 min was sufficient for reducing participants’ sexuality myth endorsement. Future research can explore whether other metrics of adoption—like the amount of time individuals spend viewing posts on Instagram—are potentially more relevant to the extent that individuals benefit from sexuality social media knowledge translation initiatives than public engagement with initiatives.

Our findings with respect to penetration and the small but positive effect of MisconSEXions content on participants’ myth endorsement shed light on the potential importance of repeated or prolonged exposure to sexuality social media knowledge translation for enduring sexual attitude change. Participants perceived that exposure to MisconSEXions content “somewhat” changed their attitudes about topics discussed in the posts and only changed their attitudes about sexuality in general “a little bit.” Potentially, for individuals to perceive that their overall sexual attitudes changed, they may require longer and repeated exposure to MisconSEXions content, as opposed to a brief, single exposure (approximately 5 min on average) in our study. Indeed, research on general attitude change suggests that shifts in attitudes occur gradually over time through repeated exposure to new information (Olson and Zanna, 1993; Ranganath and Nosek, 2008; Bohner and Dickel, 2011; Benton et al., 2022). Given that participants expressed interest in learning more about sexuality after viewing MisconSEXions content, they may be motivated to repeatedly view the same or new content. This highlights a potential strength of delivering MisconSEXions content via Instagram; repeated exposure to MisconSEXions content on Instagram may facilitate larger and enduring change in individuals’ overall sexual attitudes.

### 4.3 Strengths, limitations, and future directions

Our study boasts a large and inclusive sample with respect to gender modality, sexual orientation, and race/ethnicity/nationality. Indeed, nearly 50% of the sample reported a minoritized sexual orientation and over 10% of the sample reported being transgender or gender-diverse. This diversity allowed us to examine group differences in individuals’ experience of MisconSEXions content, providing important insight into ways that groups do or do not differ with respect to the impact and their perceptions of the social media knowledge translation initiative. The diversity of the sample in terms of gender modality and sexual orientation is particularly important given that most sex education curricula and sex research studies predominantly focus on majoritized identities (Steinke et al., 2017; Rabbitte, 2020; Garg and Volerman, 2021; Pampati et al., 2021; Delmonaco and Haimson, 2022; Jia et al., 2022; Epps et al., 2023).

The design of our study is limited in terms of external validity because we required participants to view MisconSEXions content within a survey platform rather than directly on Instagram. Therefore, while the present study represents an important first step in providing evidence about the impact of MisconSEXions content and participants’ perceptions of their hypothetical engagement with MisconSEXions content on Instagram, we were unable to examine knowledge change and implementation outcomes when MisconSEXions is used on Instagram itself. This is an important limitation given that individuals’ intentions to act often fail to translate into actual behaviors (Sheeran and Webb, 2016), which is relevant for the adoption outcomes. Indeed, with respect to adoption in the present study, despite reporting low hypothetical intentions to engage with MisconSEXions, participants spent over 5 min viewing MisconSEXions content on average. Future research should assess the effectiveness and implementation outcomes of sexuality social media knowledge translation initiatives in more ecologically valid ways, including behavioral measures of implementation (e.g., actual “liking” and sharing of posts). This will provide an understanding of how individuals engage with initiatives outside of the context of a survey and whether ongoing exposure to MisconSEXions content on Instagram produces even greater reductions in sexuality myth endorsement.

## 5 Conclusion

The results of the present study provide preliminary evidence that a sexuality social media knowledge translation initiative—MisconSEXions—reduces individuals’ general sexuality myth endorsement regardless of their assigned sex, gender modality, or sexual orientation. Our findings expand upon recent studies demonstrating that social media knowledge translation initiatives are acceptable and bolster sexual knowledge about *specific* sexual problems or health (Bull et al., 2012; Guse et al., 2012; Gabarron and Wynn, 2016; Stevens et al., 2017; Wadham et al., 2019; Beischel et al., 2021; Rosen et al., 2021; Jackowich et al., 2022) by demonstrating that MisconSEXions is an acceptable initiative that reduces *general* sexuality myth endorsement. The results for the implementation outcomes highlight that intentional efforts to develop inclusive sex education can result in initiatives that appeal broadly across assigned sex, gender modality, and sexual orientation groups. Overall, social media knowledge translation initiatives may be one novel way to fill important gaps in sex education curricula. Future research should further examine the implementation of sexuality knowledge translation initiatives when accessed on social media rather than within a survey platform. Researchers can use our findings to inform the development of sexuality social media knowledge translation initiatives that are impactful, inclusive, and appealing to diverse groups.

## Data availability statement

The original contributions presented in this study are included in this article/supplementary material, further inquiries can be directed to the corresponding author.

## Ethics statement

The studies involving humans were approved by the University of British Columbia Behavioural Research Ethics Board. The studies were conducted in accordance with the local legislation and institutional requirements. The participants provided their written informed consent to participate in this study.

## Author contributions

KO’K: Conceptualization, Formal analysis, Investigation, Writing – original draft, Writing – review & editing. SG: Formal analysis, Investigation, Writing – review & editing. KB: Investigation, Supervision, Writing – original draft, Writing – review & editing. SD: Conceptualization, Data curation, Investigation, Methodology, Supervision, Writing – original draft, Writing – review & editing.

## Footnotes

1 Gender identity refers to an individual’s internal sense of their gender, while gender modality refers to the congruence or incongruence between an individual’s current gender identity and the gender/sex they were assigned at birth. An individual’s gender identity may include identities like woman, man, non-binary, etc., whereas their gender modality may be cisgender or transgender and gender-diverse. We use the term gender modality throughout this paper to capture the unique experiences transgender and gender-diverse people have relative to cisgender people in regard to sexual knowledge, given that transgender and gender-diverse individuals are more likely to experience barriers to accessing information about sexuality relevant to their identities and bodies than cisgender individuals.

2
https://osf.io/pj75v/
